# Supplementary material for: Exploring the connection between pet attachment and owner mental health: The roles of owner-pet compatibility, perceived pet welfare, and behavioral issues
Source: PLoS One. 2025 Oct 14;20(10):e0314893. doi: 10.1371/journal.pone.0314893 (PMC12520413; doi:10.1371/journal.pone.0314893)
Supplement: S1 Table — (DOCX) [file pone.0314893.s001.docx]

**S1 Table.** Linear regressions for insecure pet attachment (IV) predicting owner mental health severity (DV).

| **Dog owner anxious attachment** | | | | | | |
| --- | --- | --- | --- | --- | --- | --- |
|  | R | R^2^ | F | df | p | β |
| Anxiety | .13 | .02 | 5.85 | 1,340 | **.016** | .72 |
| Depression | .20 | .04 | 14.50 | 1,340 | **<.001** | 1.34 |
| **Dog owner avoidant attachment** | | | | | | |
|  | R | R^2^ | F | df | p | β |
| Anxiety | .10 | .01 | 3.49 | 1,340 | .063 | -.57 |
| Depression | .002 | .00 | .001 | 1,340 | .98 | -.01 |
| **Cat owner anxious attachment** | | | | | | |
|  | R | R^2^ | F | df | p | β |
| Anxiety | .01 | .00 | .01 | 1,258 | .938 | .02 |
| Depression | .03 | .00 | .16 | 1,258 | .689 | -.15 |
| **Cat owner avoidant attachment** | | | | | | |
|  | R | R^2^ | F | df | p | β |
| Anxiety | .15 | .02 | 6.13 | 1,258 | .**014** | -.76 |
| Depression | .16 | .03 | 7.06 | 1,258 | **.008** | -1.09 |

*Notes***:** R = simple correlation, R^2^= total variation in DV explained by IV. Regression coefficients (β) are standardized, representing the expected change in the dependent variable (in SD units) for a one‑SD change in each predictor, therefore indicating the strength and direction of the predictor variables’ impact on the dependent variable. All inferential tests were two-tailed with significance set at *p* < .05.
