# Supplementary material for: Exploring the connection between pet attachment and owner mental health: The roles of owner-pet compatibility, perceived pet welfare, and behavioral issues
Source: PLoS One. 2025 Oct 14;20(10):e0314893. doi: 10.1371/journal.pone.0314893 (PMC12520413; doi:10.1371/journal.pone.0314893)
Supplement: S2 Table — (DOCX) [file pone.0314893.s002.docx]

**S2 Table.** Spearman two-tailed correlations between pet attachment and perceived dog welfare, and between perceived dog welfare and owner mental health.

|  | 1 | 2 | 3 | 4 | 5 | 6 | 7 | 8 | 9 | 10 |
| --- | --- | --- | --- | --- | --- | --- | --- | --- | --- | --- |
| 1 Avoidant attachment | 1 | .371** | **-.231**** | **-.182**** | **-.258**** | **-.232**** | **-.295**** | **-.139*** | -.034 | **-.119*** |
| 2 Anxious attachment |  | 1 | **-.405**** | **-.231**** | **-.272**** | **-.242**** | **-.354**** | **-.219**** | **.196**** | **.124*** |
| 3 Dog happiness |  |  | 1 | .452** | .350** | .421** | .681** | .466** | -.023 | .022 |
| 4 Dog physical functioning |  |  |  | 1 | .450** | .512** | .825** | .555** | **-.112^*^** | -.060 |
| 5 Dog hygiene |  |  |  |  | 1 | .405** | .711** | .399** | -.007 | -.028 |
| 6 Dog mental status |  |  |  |  |  | 1 | .761** | .448** | **-.163^**^** | **-.153^**^** |
| 7 Dog QoL total |  |  |  |  |  |  | 1 | .610** | **-.106^*^** | -.079 |
| 8 Dog welfare (direct Qol) |  |  |  |  |  |  |  | 1 | -.081 | -.028 |
| 9 Depression |  |  |  |  |  |  |  |  | 1 | .661^**^ |
| 10 Anxiety |  |  |  |  |  |  |  |  |  | 1 |

*Notes***:** Significant results of interest are in bold. ** Correlation is significant at the 0.01 level (2-tailed). * Correlation is significant at the 0.05 level (2-tailed).
