# Supplementary material for: Exploring the connection between pet attachment and owner mental health: The roles of owner-pet compatibility, perceived pet welfare, and behavioral issues
Source: PLoS One. 2025 Oct 14;20(10):e0314893. doi: 10.1371/journal.pone.0314893 (PMC12520413; doi:10.1371/journal.pone.0314893)
Supplement: S3 Table — (DOCX) [file pone.0314893.s003.docx]

**S3 Table.** Spearman two-tailed correlations between pet attachment and perceived cat welfare, and between perceived cat welfare and owner mental health.

|  | 1 | 2 | 3 | 4 | 5 | 6 | 7 | 8 |
| --- | --- | --- | --- | --- | --- | --- | --- | --- |
| 1 Avoidant attachment | 1 | .315^**^ | **-.365^**^** | **-.163^**^** | **-.294^**^** | **-.159^*^** | **-.177^**^** | **-.190^**^** |
| 2 Anxious attachment |  | 1 | **-.385^**^** | **-.275^**^** | **-.375^**^** | **-.321^**^** | -.034 | -.008 |
| 3 Cat healthy behaviors |  |  | 1 | **.381^**^** | **.791^**^** | **.387^**^** | .093 | .096 |
| 4 Cat clinical signs |  |  |  | 1 | **.793^**^** | **.406^**^** | -.018 | .007 |
| 5 Cat QoL total |  |  |  |  | 1 | **.478^**^** | .024 | .039 |
| 6 Cat welfare (direct QoL assessment) |  |  |  |  |  | 1 | .045 | **.132^*^** |
| 7 Depression |  |  |  |  |  |  | 1 | .715^**^ |
| 8 Anxiety |  |  |  |  |  |  |  | 1 |

*Notes:* significant results of interest are in bold. ** Correlation is significant at the 0.01 level (2-tailed). * Correlation is significant at the 0.05 level (2-tailed).
