# Supplementary material for: Exploring the connection between pet attachment and owner mental health: The roles of owner-pet compatibility, perceived pet welfare, and behavioral issues
Source: PLoS One. 2025 Oct 14;20(10):e0314893. doi: 10.1371/journal.pone.0314893 (PMC12520413; doi:10.1371/journal.pone.0314893)
Supplement: S4 Table — (DOCX) [file pone.0314893.s004.docx]

**S4 Table.** Parallel mediation analysis examining a) indirect effects of anxious owner-dog attachment (X) on depression symptom severity (Y), via dogs physical functioning (M), and b) indirect effects of avoidant owner-dog attachment (X) on anxiety symptom severity (Y), via dogs physical functioning (M).

|  | Indirect effects of anxious owner-dog attachment (X) on depression symptom severity (Y), via dogs physical functioning (M). | | | Indirect effects of avoidant owner-dog attachment (X) on anxiety symptom severity (Y), via dogs physical functioning (M). | | |
| --- | --- | --- | --- | --- | --- | --- |
|  | β | SE | 95% CI | β | SE | 95% CI |
| Completely standardised indirect effect beta values of X on Y (ab_cs_) (total) | .016 | .012 | -.005, .040 | .018 | .012 | -.001, .044 |
| Direct effect of X on M (a1) | -.149* | .039 | -.225, -.073 | -.142* | .040 | -.220, -.064 |
| Direct effect of M on Y (b1) | -.703 | .494 | -1.674, .268 | -.708 | .420 | -1.530, .115 |
| Direct effect of X on Y (c`) | 1.236* | .359 | .529, 1.943 | -.670* | .310 | -1.280, -.060 |
| Indirect effect of X on Y via M | .105 | .077 | -.036, .271 | .101 | .066 | -.005, .247 |

*Notes:* * Significant pathway (p < 0.05). Effect sizes: abcs = 0.01 (small effect), abcs = 0.09 (medium effect), and abcs = 0.25 (large effect). M= dogs physical functioning.
