# Supplementary material for: Exploring the connection between pet attachment and owner mental health: The roles of owner-pet compatibility, perceived pet welfare, and behavioral issues
Source: PLoS One. 2025 Oct 14;20(10):e0314893. doi: 10.1371/journal.pone.0314893 (PMC12520413; doi:10.1371/journal.pone.0314893)
Supplement: S5 Table — (DOCX) [file pone.0314893.s005.docx]

**S5 Table.** Mediation analysis examining a) indirect effects of anxious owner-dog attachment (X) on depression symptom severity (Y), via dogs total quality of life scores (CHQLS) (M), and b) indirect effects of avoidant owner-cat attachment (X) on anxiety symptom severity (Y), via cats quality of life scores (direct assessment) (M).

|  | Indirect effects of anxious owner-dog attachment (X) on depression symptom severity (Y), via dogs total quality of life scores (CHQLS) (M). | | | Indirect effects of avoidant owner-cat attachment (X) on anxiety symptom severity (Y), via cats quality of life scores (direct assessment) (M). | | |
| --- | --- | --- | --- | --- | --- | --- |
|  | β | SE | 95% CI | β | SE | 95% CI |
| Completely standardised indirect effect beta values of X on Y (ab_cs_) (total) | .018 | .018 | -.019, .053 | .018 | .022 | -.023, .063 |
| Direct effect of X on M (a1) | -.160* | .026 | -.212, -.108 | -3.080* | .562 | -4.186, -1.974 |
| Direct effect of M on Y (b1) | -.763 | .728 | -2.195, .699 | -.029 | .034 | -.096, .039 |
| Direct effect of X on Y (c`) | 1.219* | .371 | .489, 1.948 | -.853* | .326 | -1.495, -.211 |
| Indirect effect of X on Y via M | .122 | .121 | -.127, .356 | .089 | .107 | -.117, .313 |

*Notes:* * Significant pathway (p < 0.05). Effect sizes: abcs = 0.01 (small effect), abcs = 0.09 (medium effect), and abcs = 0.25 (large effect). M= pet quality of life.
