# Supplementary material for: Exploring the connection between pet attachment and owner mental health: The roles of owner-pet compatibility, perceived pet welfare, and behavioral issues
Source: PLoS One. 2025 Oct 14;20(10):e0314893. doi: 10.1371/journal.pone.0314893 (PMC12520413; doi:10.1371/journal.pone.0314893)
Supplement: S6 Table — (DOCX) [file pone.0314893.s006.docx]

**S6 Table.** Relationships between owner-pet attachment, perceived pet behavioral problems, and owner mental health.

|  |  | Avoidant attachment | Anxious attachment | Owner depression | Owner anxiety |
| --- | --- | --- | --- | --- | --- |
| Dog owners | Excitability | -.147** | -.107* | -0.014 | 0.033 |
|  | Aggression | .189** | .182** | 0.078 | 0.043 |
|  | Fear & Anxiety | 0.012 | .157** | .165** | .146** |
|  | Separation issues | 0.037 | .190** | 0.085 | 0.063 |
|  | Attachment related issues | -.204** | -0.099 | 0.088 | 0.093 |
|  | Training difficulty | .160** | .238** | .146** | 0.041 |
|  | Misc | 0.106 | .133* | 0.063 | 0.036 |
|  | Total issues | 0.032 | .148* | 0.067 | 0.075 |
| Cat owners | Cat behavioral problems (total) | 0.005 | .123* | -0.012 | -0.01 |
|  | Ratings of being ‘bothered’ by problems | 0.308 | .409* | 0.158 | 0.013 |

*Notes*: significant results of interest are in bold. ** Correlation is significant at the 0.01 level (2-tailed). * Correlation is significant at the 0.05 level (2-tailed).
