# Supplementary material for: Exploring the connection between pet attachment and owner mental health: The roles of owner-pet compatibility, perceived pet welfare, and behavioral issues
Source: PLoS One. 2025 Oct 14;20(10):e0314893. doi: 10.1371/journal.pone.0314893 (PMC12520413; doi:10.1371/journal.pone.0314893)
Supplement: S7 Table — (DOCX) [file pone.0314893.s007.docx]

**S7 Table.** Relationships between owner-pet attachment and owner-pet compatibility (subscales and total score), and between owner-pet compatibility and owner mental health.

| Dog owners | Physical | Social | Affection | Closeness | Other | Total |
| --- | --- | --- | --- | --- | --- | --- |
| Owner-dog attachment avoidance | -.098 | **-.129*** | **-.325**** | **-.375**** | **-.207**** | **-.382**** |
| Owner-dog attachment anxiety | **-.158**** | **-.086** | **-.269**** | **-.285**** | **-.107*** | **-.308**** |
| Owner depression | -.045 | -.022 | .065 | -.023 | .026 | .001 |
| Owner anxiety | -.029 | -.081 | **.149**** | .074 | .075 | .065 |
| Cat owners | Physical | Social | Affection | Closeness | Other | Total |
| Owner-cat attachment avoidance | .052 | -.052 | **-.277**** | **-.252**** | -.086 | **-.246**** |
| Owner-cat attachment anxiety | -.044 | **-.225**** | **-.246**** | **-.273**** | -.035 | **-.306**** |
| Owner depression | .095 | .021 | .0001 | .113 | .957 | .197 |
| Owner anxiety | .028 | .014 | -.008 | .113 | .026 | .062 |

*Notes:* significant results of interest are in bold. ** Correlation is significant at the 0.01 level (2-tailed). * Correlation is significant at the 0.05 level (2-tailed).
