# Supplementary material for: Exploring the connection between pet attachment and owner mental health: The roles of owner-pet compatibility, perceived pet welfare, and behavioral issues
Source: PLoS One. 2025 Oct 14;20(10):e0314893. doi: 10.1371/journal.pone.0314893 (PMC12520413; doi:10.1371/journal.pone.0314893)
Supplement: Attachment — Submitted filename: PLOS R_R review pet ownership.docx [file pone.0314893.s009.docx]

I acknowledged that the manuscript has significantly improved, however, it still struggles to present the research questions with clarity and precision.

Specifically, the introduction is still too long, the research questions are repetitive, and predictions are missing. It appears the authors inadvertently introduced this issue in an effort to address my comments regarding one-to-one correspondence between research hypotheses and results. Perhaps, I can make some suggestions- this issue alone does not preclude publication.

The authors are asking

1) Are there differences between dog and cat owners on measures of pet attachment and mental health?, 2) Does insecure pet attachment relate to owner mental health symptom severity?, 3) Does perceived pet welfare explain the relationship between insecure pet attachment and owner mental health symptom severity?, 4) Do perceived pet behavioral problems explain the relationship between insecure attachment and owner mental health symptom severity?, and 5) Does owner-pet compatibility explain the relationship between insecure attachment and owner mental health symptom severity?

How about presenting it in the following manner:

1) Are there differences between dog and cat owners on measures of pet attachment and mental health?
2) Do insecure pet attachment, perceived pet welfare and behavioral problems, and owner-pet compatibility explain the relationship between insecure attachment and owner mental health symptom severity?

This will also leave the breakdown in the results section perfectly fine.

The authors should then introduce these questions around line 150, right after …present research… The rest would constitute the rationale for predictions, which are missing by the way. Where are the predictions following each research question?

In sum, introduce the significance of the study, articulate the gap in our current knowledge, present the specific research questions, and follow each with predictions grounded in a clear rationale supported by prior research.

A minor issue: In discussion, this sentence: “*These aspects, which are under-researched, are believed to play crucial roles in shaping owner-pet relationships and consequently influence owner mental well-being*” (Line 645-47) requires references. Additionally, consider revising it to use active voice rather than passive.
