# Supplementary material for: Exploring the connection between pet attachment and owner mental health: The roles of owner-pet compatibility, perceived pet welfare, and behavioral issues
Source: PLoS One. 2025 Oct 14;20(10):e0314893. doi: 10.1371/journal.pone.0314893 (PMC12520413; doi:10.1371/journal.pone.0314893)
Supplement: Attachment — Submitted filename: Response_to_Reviewers_auresp_2.docx [file pone.0314893.s011.docx]

**Response to Editor and Reviewers (15/09/25)**

**Editor**

**Comment:** The edits and revisions have substantially improved the overall quality of the manuscript. However, both the reviewers and I feel that the Introduction and Strengths and Limitations sections remain somewhat lengthy and could be streamlined to avoid verbosity. Please address the following detailed comments provided by the two reviewers.

**Response:** Thank you for the opportunity to re-submit our manuscript, we have addressed these final comments and hope you feel the manuscript is ready for publication. We have now made both the Introduction and Strengths and Limitations sections more succinct.

**Reviewer #1**

**Comment:** The authors have done a remarkable job in improving the manuscript. The introduction is still on the long side, but understandable. The discussion is a lot clearer and more nuanced, though some of the paragraphs would benefit from more conciseness and alternative explanations. I note a few minor points below and would be happy to see this important work published once these are addressed.

**Response:** Thank you for your positive feedback. In line with both reviewer’s and editors’ feedback, we have further trimmed down the Introduction to make this more succinct, as well as the Strengths and Limitations sections to be more concise, and have addressed your final feedback comments below.

**Comments:**

Abstract

L48 delete comma

L53 „Attachment notably influenced mental health” I find this phrasing still too causal given the correlative results. It has been amended well in the rest of the sentence, but this part could use a more nuanced version as well to not misguide the reader.

L61 “Owner-dog compatibility, particularly in the affection domain, influenced owner anxiety” same as above.

**Response:** These edits have now been addressed.

Introduction

**Comment:** L141 putting “negative outcomes” in brackets rather than integrated it in a sentence makes it a bit awkward to read. If possible, I would suggest integrating it as a normal part of the sentence.

**Response:** This has been re-worded.

**Comment:** L154 ff this paragraph still feels rather repetitive and could be a lot more concise.

**Response:** This paragraph has been edited to be more concise.

**Comments:**

Methods

L264 something is off in this sentence (Prolific appears twice)

L329 and L341: delete “is”

**Response:** These edits have now been addressed.

Results

**Comment:** L430 f It is unclear what these outcomes compare to. More likely than dog owners, or more likely to display insecure than secure attachment?

**Response:** More likely than dog owners - this has been re-worded for clarity.

**Comment:** L481 delete the comma before subscales?

**Response:** Have re-worded this sentence as we meant subscales of the FHQLS, and not the direct quality of life scale.

**Comment:** L480ff these outcomes are surprising. A negative relationship between insecure attachment and cat healthy behaviours but also a negative relationship between insecure attachment and cat clinical signals? How could this be explained?

**Response:** Yes, you are right that insecure attachment led to perceptions of poorer cat welfare, yet fewer clinical signs of health issues. This may highlight the difference between subjective welfare/quality of life reports and owner-reported clinical signs. Perceptions of welfare are inherently subjective and require interpretation of the cat’s emotional state, satisfaction, or “happiness.” Owners with insecure attachments may have a negative cognitive bias that influences these judgments (explained further in the discussion section). Clinical signs, in contrast, are more objective (e.g., vomiting, limping, coughing) and less open to interpretive bias. Even if insecurely attached owners evaluate welfare more negatively, they may not report increased clinical signs because those are observable, concrete events rather than perceptions. **We have now added a sentence to this effect in the discussion.**

**Comments:**

L543 closing bracket is missing

L605 two periods

**Response:** Edited.

Discussion

**Comment:** L645 you write under researched without a hyphen in the abstract

**Response:** Edited.

**Comment:** L645 “are believed” by whom? Did you hypothesise that or can you accredit this assumption to someone?

**Response:** Edited.

**Comment:** L761 I am unsure what the “or as not” is meant to express. Perhaps delete the comma afterwards?

**Response:** Edited.

**Comment:** L769 ff higher total quality of life and physical functioning relate to the pet, correct? I would suggest adding “dogs’ ” in front of the terms to help the reader stay on track with all the different variables and associations. The same goes for the rest of the paragraph. It is at times difficult to follow when a term relates to the dog or the owner without having to go back to the exact analysis.

**Response:** Thank you for this suggestion – this has now been edited for clarity throughout.

**Comment:** L831 “These findings indicate that insecurity within human-pet attachments could influence feelings of owner compatibility and thereby perceptions of the human-pet relationship”. Or the other way around (which I would personally find a more straight forward explanation)? Feeling incompatible may lead to insecure attachment – again considering what kind of demographic (adolescents at home that might not have chosen the pet/ have a thorough attachment)

**Response:** Thank you for this insightful comment; we agree that the direction could plausibly work both ways and we have clarified in the discussion that given the cross-sectional mediation design, causality cannot be determined and the relationship between insecure attachment and perceived compatibility is likely bidirectional.

**Comment:** L856 the strength and limitation section is rather long. I believe parts of that could be significantly more succinct. For example, L856 – 871: the strength could be summarized in one sentence, since this has already been discussed in the intro. Sentence 868 is not needed here, it fits better in the conclusion.

**Response:** We have now edited this section to be more concise.

Comment: L919 in the table you did not at the Q to LGBTQI+

**Response:** I could see the Q in the table (demographics); however I did notice we missed the Q on a separate mention so this has been edited.

**Comment:** Graphs: The graphs should be higher quality; they are hard to read.

**Response:** Thank you – we have tried to improve the quality of these, increasing the KBs of each.

**Reviewer #2:**

**Comments:**

I acknowledged that the manuscript has significantly improved, however, it still struggles to present the research questions with clarity and precision. Specifically, the introduction is still too long, the research questions are repetitive, and predictions are missing. It appears the authors inadvertently introduced this issue in an effort to address my comments regarding one-to-one correspondence between research hypotheses and results. Perhaps, I can make some suggestions- this issue alone does not preclude publication. The authors are asking 1) Are there differences between dog and cat owners on measures of pet attachment and mental health?, 2) Does insecure pet attachment relate to owner mental health symptom severity?, 3) Does perceived pet welfare explain the relationship between insecure pet attachment and owner mental health symptom severity?, 4) Do perceived pet behavioral problems explain the relationship between insecure attachment and owner mental health symptom severity?, and 5) Does owner-pet compatibility explain the relationship between insecure attachment and owner mental health symptom severity? How about presenting it in the following manner: 1) Are there differences between dog and cat owners on measures of pet attachment and mental health?, 2) Do insecure pet attachment, perceived pet welfare and behavioral problems, and owner-pet compatibility explain the relationship between insecure attachment and owner mental health symptom severity?

This will also leave the breakdown in the results section perfectly fine. The authors should then introduce these questions around line 150, right after …present research… The rest would constitute the rationale for predictions, which are missing by the way. Where are the predictions following each research question? In sum, introduce the significance of the study, articulate the gap in our current knowledge, present the specific research questions, and follow each with predictions grounded in a clear rationale supported by prior research.

**Response:** We really appreciate the time and effort you have put in to help us improve the manuscript – we have taken on your suggestions and have re-worded/ordered the research questions, and included the predictions for each. We have cited theory and research to support each prediction, but did not want to repeat the rationales provided throughout the introduction section. We have kept the order and flow of the results the same but have removed the research question numbering. We hope this has made the flow clearer and avoids repetition. We have also now made the introduction, and some sections of the discussions more succinct in line with the other reviews.

**Comment:** A minor issue: In discussion, this sentence: “These aspects, which are under-researched, are believed to play crucial roles in shaping owner-pet relationships and consequently influence owner mental well-being” (Line 645-47) requires references. Additionally, consider revising it to use active voice rather than passive.

**Response:** This has been edited, and relevant citations have been included to support this statement.
